# Mental Health in Academia: The Role of Workplace Relationships

**DOI:** 10.3389/fpsyg.2020.562457

**Published:** 2020-09-02

**Authors:** Thiago C. Moulin

**Affiliations:** ^1^Department of Neuroscience, Uppsala University, Uppsala, Sweden; ^2^Institute of Medical Biochemistry Leopoldo de Meis, Federal University of Rio de Janeiro, Rio de Janeiro, Brazil

**Keywords:** academia, mental health, relationships, biomedical research, workplace

## Introduction

Discussions about mental health in academia have painted a concerning picture. A recent online survey with 2,279 participants from 26 countries and 234 institutions reported that graduate students are more than six times as likely to experience depression and anxiety as compared to the general population (Evans et al., 2018). Similarly, a study evaluating mental health problems among 3,659 Ph.D. students from Belgium indicates that the prevalence of psychiatric disorders is higher among academics than the general highly educated population (Levecque et al., 2017). Additionally, when directly screened for depressive symptoms, 47% of Ph.D. students and 37% of Master's students surveyed at the University of California met the required diagnosis score for depressive disorder (Assembly, 2014).

However, some studies on job satisfaction in the research community somewhat contradict the concerns regarding academic well-being. A survey among pharmacy school faculties in the U.S. with 811 participants showed that 86.3% of respondents stated a desire to stay in academia (Lindfelt et al., 2018). Moreover, 63.7% of the faculty members reported being very or extremely satisfied with their current academic position (Lindfelt et al., 2015). Amid Ph.D. students, an engagement survey from the University of Helsinki indicated that only 33% of the respondents had considered dropping out, which is lower than the reported 43% withdrawal intention for the whole university (Sakurai et al., 2012). Lastly, a recent worldwide survey from *Nature* on the career aims and struggles of around 5,700 Ph.D. students showed that, although more than one-quarter of the respondents were concerned about mental health, about 80% were satisfied or very satisfied with their decision to do a Ph.D. (Woolston, 2017).

In this article, I argue how the relationships between colleagues and supervisors in academic environments can be a central factor for understanding these seemingly conflicting data. Then, based on personal experience and published literature, I discuss how the unique work situations of biomedical research influence interpersonal relations and how it bidirectionally links to job satisfaction.

## Main Text

Spending years in academia has allowed me to connect with a broad range of biomedical researchers. After various conferences, lectures, and meetings, it was impossible not to admire the researchers confidently speaking about their groundbreaking findings. As an early student, falling in love with such a welcoming and beautiful science was unavoidable. However, it was by talking to other graduate students that a less glamorous side could be witnessed: advisors that cared about quantity rather than quality and placed undue pressure on their students to increase numbers; professors that wanted to nurture their egos at other people's expense; absent tutors that did not remember what they had discussed with their students the day before (or sometimes, did not even recall their projects). As a Ph.D. student, I realized that having proper guidance was hard, and I was lucky. In fact, although overall satisfied with their academic path, recent reports show that fewer than 40% of Ph.D. students are happy with their tutoring. Moreover, 23% said they would change advisors if possible, and another 18% said that they do not have useful conversations about careers with them (Woolston, 2017). The picture remains equally unsettling after becoming an early-career postdoc. The constant pressure for fund gathering and high productivity, plus the responsibilities of being a faculty member, create a notoriously overwhelming situation (Lashuel, 2020). Yes, without guidance or social support, academia can feel like a lonely place.

However, in this seemingly inhospitable environment, it is possible to come across many honest and healthy relationships. The popular saying, despite worn out, sounds correct: goodness thrives when times are tough. Across a range of different laboratories and research groups and through their unique dynamics, it is usual to find friendships growing through the shared burden of long hours of work. Importantly, evidence shows that these close workplace relationships can improve well-being, as variables like colleague belongingness and positive relationships with co-workers are significant predictors of self-reported health (Persson et al., 2018). A positive atmosphere, as such, would be especially significant in biomedical research, where everyday chores may involve sacrifices like laborious day-long experiments, overnight shifts, busy weekends, and periods of intense stress due to deadlines (Landrigan et al., 2006; Thomsen et al., 2006). It is not surprising that support and understanding, which is necessary for any relationship, can be easily found among those with whom these challenges are shared.

Accordingly, case reports indicate that support from colleagues and superiors is essential for dealing with mental health struggles in academia (Loissel, 2019). Graduate students who had low scores in questionnaires for anxiety or depressive disorder were more likely to have a positive evaluation of their relationship with their principal investigators or supervisors (Evans et al., 2018). By providing early psychosocial help, the mentoring support found in the lab environment contributes to the students' well-being (Tenenbaum et al., 2001) and can make a difference in the fight against mental disorders, even before a formal diagnosis. Indeed, laboratory colleagues and group members are, in many cases, the few people close enough to someone showing initial signs of distress, and may be able to notice subtle symptoms, like apathy or restlessness (Loissel, 2019).

Additionally, it is not uncommon to find researchers who have or had romantic partners within academia, who often are collaborators or members of the same laboratory. Many institutions have policies to avoid such relationships in supervisory situations, which is strongly supported by faculty members (Bellas and Gossett, 2001). Nevertheless, unlike many other work environments (Kolesnikova and Analoui, 2012), where hierarchy-dependent positions are respected, romantic relationships are widely accepted within academia. In fact, a survey among graduate students provides evidence that participating in a romantic relationship with a member of the same organization was positively associated with self-appraised job performance and intrinsic work motivation (Pierce, 1998). From personal experience, having a partner within a similar research field has helped to improve numerous manuscripts and presentations due to the overlook of a caring pair of fresh eyes. Most importantly, when feeling overwhelmed and in need of mental support, I could count on the help of someone that would understand the problem. Of course, each case is unique, but over the years, the act of sharing concerns and rewards of research endeavors brought me closer to the people I cared the most.

Considering these observations altogether, I believe that better recognition should be given to those who provide much-needed support throughout the arduous academic path. Independent of the type of relationship, from bench colleagues to advisors, those that are kind to us must be esteemed. If we encourage a positive environment, a culture of friendship and respect may grow, making research groups, and institutions a better place to work. When feeling valued, people enjoy the work and, ultimately, produce better science.

## Conclusions

It is essential to distinguish that, although acknowledging that academic research is an intrinsically hard path, we cannot allow abusive workplaces. There is a line between challenging environments and displeasing ones — and where to draw the line is an individual's own decision. Mental health is definitely a concerning issue in academia, and we should pay closer attention to each other's struggles.

Nevertheless, we should not forget to cherish the good people around us. By appreciating the ones that make day-to-day research lighter, it is possible to nurture constructive and encouraging workplaces. After all, academic thinking works much better when we are thoughtful.

## Author Contributions

The author confirms being the sole contributor of this work and has approved it for publication.

## Conflict of Interest

The author declares that the research was conducted in the absence of any commercial or financial relationships that could be construed as a potential conflict of interest.
